# Miswired Proprioception in Amyotrophic Lateral Sclerosis in Relation to Pain Sensation (and in Delayed Onset Muscle Soreness)—Is Piezo2 Channelopathy a Principal Transcription Activator in Proprioceptive Terminals Besides Being the Potential Primary Damage?

**DOI:** 10.3390/life13030657

**Published:** 2023-02-27

**Authors:** Balázs Sonkodi

**Affiliations:** Department of Health Sciences and Sport Medicine, Hungarian University of Sports Science, 1123 Budapest, Hungary; bsonkodi@gmail.com

**Keywords:** amyotrophic lateral sclerosis, Piezo2 channelopathy, transcription, pain, WDR neuron, aging

## Abstract

Amyotrophic lateral sclerosis (ALS) is a lethal neurodegenerative multisystem disease, with an unknown pathomechanism, resulting in progressive motoneuron loss. In 90–95% of cases, ALS is sporadic, but close to 10% of ALS is familial with inherited gene mutations from family members. Recently, a non-contact dying-back injury mechanism theory of ALS postulated that irreversible intrafusal proprioceptive terminal degeneration induces the non-resolving progressive impairment of the proprioceptive circuitry, leading to motoneuron loss, progressive overloading and depletion of the central nervous system, and eventually to death. The current manuscript proposes that irreversible Piezo2 channelopathy of this proprioceptive terminal degeneration induces constantly activated and dysregulated transcription process in ALS, providing access to underlying pathogenic gene variants and letting the cell-type-specific noncoding DNA mutations become more apparent. This opinion piece proposes that ALS genes are associated with the Piezo2 channelopathy mechanism both downstream and upstream, and their mutations, along with the aging process, could explain the non-contact dying-back injury mechanism theory of ALS. Moreover, irreversible microinjury of the Piezo2 ion channel could be the primary damage or the root cause of death in ALS. Finally, the current manuscript also depicts the pathomechanism as to why ALS is considered a painless disease.

## 1. Introduction

Amyotrophic lateral sclerosis (ALS) is a lethal neurodegenerative multisystem disease, with an unknown pathomechanism, resulting in progressive motoneuron loss. In around 90% of the cases, ALS is sporadic, but close to 10% of ALS is familial with inherited gene mutations from family members.

There is emerging research that sensory involvement with sensory circuit dysfunction is evident, even prior to motoneuron involvement, in the ALS disease process [1,2,3]. Correspondingly, a recent non-contact dying-back injury mechanism theory of ALS postulated that intrafusal proprioceptive terminal degeneration induces the non-resolving progressive impairment of the proprioceptive circuitry, leading to motoneuron death, progressive overloading and depletion of the central nervous system (CNS), and eventually to death [4]. Moreover, the lost function of Piezo2 ion channels on intrafusal proprioceptive terminals was implicated as the root cause of death and the loci of the primary damage leading to it in ALS [5].

Ardem Patapoutian, a Nobel Prize laureate, and his team demonstrated that Piezo2 is the principal ion channel responsible for the mechanotransduction of proprioception [6]. It has been theorized that these Piezo2 ion channels could be microinjured in the proprioceptive terminals of the muscle spindle under repetitive fatiguing due to forced longitudinal contractions inducing an allostatic stress time window [7,8,9]. Furthermore, Fernandez-Trillo et al. emphasized that Piezo2 containing somatosensory fibers carry special genetic signatures [10]. In addition, the importance of the Piezo2 containing intrafusal proprioceptive fibers is highlighted because their terminal microinjury could lead to encroachment of our most profound life-sustaining genetic encoding through the impairment of the stretch reflexes [5,8,11]. The potential role of these proprioceptive fibers in growth, regeneration, and even remodeling has been suggested [12]. The current author suggests that the primary microinjury of Piezo2 on proprioceptive terminals could activate transcription pathways that are the basis for the aforementioned growth, regeneration, and remodeling processes. However, the lost function of Piezo2 results not only the painless feature of ALS, but it is also incompatible with life sustainment.

It is important to note that it could be learnt from chemotherapy that these types of intrafusal proprioceptive terminals could go through non-Wallerian-like axon terminal degeneration in an acute and chronic fashion, as well in a dose limiting manner [13,14]. Accordingly, it was postulated that acute microinjury of these proprioceptors could be experienced in delayed onset muscle soreness (DOMS) [12], chronic microinjury could be experienced in other non-contact injury-induced disease conditions [8], and the irreversible type of this microinjury with associated neuromuscular detachment of proprioceptors in the muscle spindle could be experienced in ALS with the involvement of environmental risk factors and genetic predisposition [4]. Correspondingly, it has been put forward that irreversibly lost function of intrafusal proprioceptive terminal Piezo2 due to a direct or indirect cause could be in conflict with maintenance of life in ALS [5].

The current paper intends to highlight some critical pathways that could lead to the irreversible loss of Piezo2 function downstream, and the consequence of it upstream, resulting in the characteristic symptoms of ALS, that eventually lead to death.

## 2. Piezo2 Channelopathy

It is important to understand the pathomechanism of the Piezo2 channelopathy theory in order to understand the pathways that could lead to this principal microinjury gateway directly or indirectly.

### 2.1. Piezo2 Ion Channels

Piezo ion channels are excitatory, nonselective, cation channels and the largest transmembrane proteins with a role in mechanosensation through force-gated excitation [15,16]. Piezo channels have an essential mechanotransductory role in homeostasis maintenance, with Piezo1 being the homeostatic gatekeepers in peripheral tissues [17,18,19,20,21,22], while Piezo2 carries the same role on somatosensory neurons and in the CNS [21,23,24]. These life sustaining signals transduced by Piezo channels are touch sensation, proprioception, and cardiovascular regulation, but they also contribute to cell alignment and shear stress detection [25]. Correspondingly, Piezo1 channels are cellular mechanoceptors in the peripheral tissues and can be neuromodulators through cross-talking with the sensory Piezo2 ion channels [26]. One more important feature of Piezo channels is that they can sense mechanotransducing signals spatially [27], providing the base for position sense and reset homeostasis on a whole body level [28,29]. Cross modulation between Piezo1 and Piezo2 is most likely indispensable for performance enhancement as well [9,26].

### 2.2. Piezo2 Channelopathy

Recently, it was hypothesized that unaccustomed or strenuous eccentric contraction-induced ASR could invoke an autologous mechano-energetic lesion of Piezo2 in intrafusal proprioceptive terminals, as is suggested in the primary injury phase of non-contact injuries, such as DOMS [8,12,30,31].

Neuro-energetically, the stable extension of the limits of homeostasis during strenuous exercise is allostasis [32]. Correspondingly, challenging these limits induces allostatic stress, which is analogous to the aforementioned ASR time window. It is noteworthy that excitation activates Piezo2, while hyperexcitation inactivates it due to prolonged muscle stretch [33,34]. Nevertheless, this hyperexcitation could cross the limits of homeostasis, therefore, two states should be differentiated, namely a physiological one where proprioceptive terminal hyperexcitation is without microdamage and Piezo2 is inactivated. In contrast is a pathophysiological one, where proprioceptor terminal hyperexcitation is associated with microdamage and could induce Piezo2 channelopathy, meaning that Piezo2 channels become leaky to subthreshold imbalanced leakage currents when they should be inactivated [8]. It is important to note that Piezo2 is a non-selective cation ion channel; however, it has a preference for Ca^2+^ [35,36]. Moreover, the underlying mitochondrial mechano-energetic deficiency could also impair glutamate vesicular release in proprioceptive terminals. Hence, microdamaged Piezo2 can not only become leaky to subthreshold imbalanced Ca^2+^ currents in the hyperexcited and supposedly inactivated state, but also to glutamate [4,8]. The analogy could come from the so-called terminal arbor degeneration (TAD)-like somatosensory terminal impairment that can be often seen in platinum analogue and paclitaxel chemotherapy [12,37]. It is noteworthy that these TAD-like lesions do not come with classic Wallerian-type axonal degeneration and evolve through a dose limiting manner [12,13,14]. Furthermore, this microdamage alters only the static phase firing sensory component of the stretch reflex and leaves the dynamic sensory encoding basically unharmed [14].

Chemotherapy-induced TAD also showed that this impairment could come in an acute and chronic fashion, as well in a dose limiting manner [13,14]. Correspondingly, it was postulated that the acute microinjury of these proprioceptors could be experienced in DOMS [12], and chronic microinjury could be experienced in other non-contact disease conditions [8]. Even more importantly, it is theorized that the irreversible type of this microinjury with associated neuromuscular detachment of proprioceptors in the muscle spindle could be experienced in ALS with genetic predisposition and the involvement of environmental risks [4].

Important to note that sudden elevated tension of surrounding membrane lipid bilayers could activate Piezo2 [38], as the largest transmembrane protein, but hyperexcitation inactivates it. Nevertheless, ASR or aging-associated elevated lipid peroxidation could microdamage these Piezo2 encompassing lipid bilayers and that could result in the structural instability of Piezo2 with the aforementioned leakage of imbalanced subthreshold cationic, mainly Ca^+^, currents and glutamate under hyperexcitation [8]. ASR and aging-derived heightened lipid peroxidation activity could also impair Piezo2 through damage of the phospholipid substrate PIP2 of myotubularin related protein-2, which is known to have a role in the control of Piezo2 dependent mechanotransduction [39]. Furthermore, the pathological hyperexcitation of primary afferent terminals could lead to disruption of membrane cholesterol organization, therefore, they could also contribute indirectly to Piezo channels’ structural and functional instability [31], as was demonstrated in reference to Piezo1 [40]. Moreover, inadequate mitochondrial trafficking and mitochondrial mechano-energetic depletion could occur due to unaccustomed or strenuous eccentric contractions in DOMS and ALS [12], as is the case in platinum analogue and paclitaxel chemotherapy [13,14].

It is also important to note that this primary phase of non-contact injuries, namely Piezo2 channelopathy, is a silent one, because pain evolves with C-fiber sensory contribution only if harsher tissue injury follows this pain-free proprioceptive Piezo2 microinjury [8,31]. Hence, the irreversible type of this microinjury with associated neuromuscular detachment of proprioceptors in the muscle spindle will remain silent, as could be the case in ALS [4] (See Table 1).

### 2.3. Piezo2 Channelopathy-Induced Activated Transcription Process

Fernandez-Trillo et al. reported that Piezo2-containing somatosensory fibers carry special genetic signatures [10]. Moreover, the significance of the Piezo2-containing proprioceptive fibers has been also highlighted because their terminal Piezo2 channelopathy could lead to encroachment of our most profound life-sustaining genetic programing through the static phase firing encoding impairment of the stretch reflexes [5,8,11]. Moreover, Ye et al. postulated that Piezo1 has a role in transcriptional control based on their observation that hyperglycemia translocates Piezo1 into the nucleus [41]. Indeed, they demonstrated that silencing Piezo1, Piezo2, or both genes resulted in the differential expression of 3292, 1656, and 1920 genes, respectively [41]. This finding is in line with a recent theory that Piezo1 channelopathy could evolve into Piezo2 channelopathy through a chronic path, further substantiating the presence of Piezo bidirectional crosstalk between Piezo1 and Piezo2 [26]. In addition, the current author theorizes that Piezo channelopathies are associated with gene transcription activation. However, Piezo2 channelopathies of proprioceptive terminals could be principal transcription activators when it comes to pathology. The regeneration or healing of these channelopathies cannot be duly fulfilled in the presence of genetic predisposition, environmental risks, or repetitive re-injury, especially when the aging process comes along [8,9]. Hence, the unfinished healing also means that an activated transcription process kept turned on due to Piezo2 microinjury, in a cell-specific way, as is suggested, for example, in dry eye disease or psoriasis [9,26]. As a result of chronic Piezo2 channelopathy, upregulation of Piezo2 in affected dorsal root ganglions (DRG) and upregulation of Piezo1 on peripheral cells, such as keratinocytes, is suspected [26]. Indicative of this theory, research is on the rise that functionally flawless Piezo ion channels behave as “molecular breaks” in the closing down of the wound healing process [42]. Moreover, healing takes longer if escorted by Piezo1 [43]. Not to mention that the participation of neurons in wound healing will ensue an even more complex wound healing process [44], highlighting the potential controlling role of Piezo2-containing somatosensory terminals in the wound healing process [26]. Consequently, Piezo2 channelopathies could enhance this break function with simultaneous transcription activation, therefore, occluding the appropriate wound closure and healing.

Even more importantly, the proposed irreversible type of this Piezo2 microinjury in ALS is steering the process towards activated and dysregulated transcription, and apoptosis of proprioceptors and motoneurons, instead of completing the transcription process. Moreover, the loss of Piezo2 functionality will also impede the theorized crosstalk between proprioceptive Piezo2 and peripheral cell Piezo1 [26]. However, the initial Piezo2 microinjury-evoked compensatory miswired microcircuits stay alive partially in ALS and lead to sensory circuit dysfunction and progressive loading of the CNS regardless of proprioceptor detachments in the muscle spindles [4]. It is noteworthy that approximately 10% of ALS is familial, but heritability is around 50% even among sporadic ALS patients [45]. It is also demonstrated that these missing heritability clues should be investigated on noncoding chromosomal loci [46,47,48]. Furthermore, the function of these noncoding DNAs is rather cell-type-specific [49]. The author of this manuscript proposes that the irreversible Piezo2 channelopathies could initially activate transcription in ALS, providing access to the underlying pathogenic gene variants and letting the cell-type-specific noncoding DNA mutations become more apparent as well. This progressive pathomechanistic process eventually leads to the irreversible functional loss of Piezo2 ion channels in the proprioceptive terminals, hence to the loss of mechanotransductory homeostasis, and finally to death.

The question rightly addressed what could be the pathways that lead to this irreversible Piezo2 terminal detachment in the proprioceptors of the muscle spindle in ALS?

## 3. Miswiring Due to Piezo2 Channelopathy

The proposed consequence of the Piezo2 channelopathy is the “mis-wiring” phenomenon often mentioned in the scientific literature in reference to chemotherapy and non-contact injuries. Onate depicted a similar phenomenon after anterior cruciate ligament reconstruction using the analogy that an inappropriately fixed electrical cord cannot conduct electricity in the prior manner [50]. Mendelsohn demonstrated that the loss of proprioceptor activity due to transmission block did not reduce the proprioceptor input into the spinal cord; however, antagonist muscle innervation was avoided [51].

It is presumed that when a sustained muscle stretch occurs, Piezo2 channels are inactivated, and from then on, voltage-gated sodium channel 1.1 (Na_v_1.1) activity is required in order to maintain regular and reliable firing [52]. Espino et al. demonstrated that Na_v_1.1 is essential in proprioceptive signaling and addressed whether the loss of these channels on proprioceptive afferents could cause the “mis-wiring” to inappropriate motor neuron pools, as shown by Mendelsohn with the avoidance of homonymous and antagonist muscles [52]. Indeed, treatment with oxaliplatin chemotherapy also impairs the ability to maintain repetitive firing during static muscle stretch in large fiber proprioceptors and riluzole, not surprisingly an ALS medication, has a selective modifying action on this proprioceptive impairment [14]. Moreover, Vincent et al. concluded that oxaliplatin causes impaired proprioception due to sensory encoding modification on muscle spindle-derived proprioceptive terminals [14]. Vincent et al. also showed, in a later investigation, that a Na^+^ blocker could induce the same proprioceptive disability [53].

The current author suggests that the proposed miswiring could have two sources, namely Piezo2 channelopathy and Na_v_1.1 channelopathy. The difference could be that Piezo2 channelopathy could be a more complex one and induces NMDA and Na^+^-persistent inward currents (PIC), as hinted earlier [4,11,31], while Na_v_1.1 or Na_v_1.6 channelopathies could evoke only NaPICs. As a consequence of these channelopathies, the latter mentioned preprogrammed compensatory pathways are built in as safety measures in order to enhance anti-gravity protection and postural control [4,5,11,31].

### 3.1. Impaired Proprioception and Delayed MLR

The aforementioned impaired glutamate vesicular release in association with Piezo2 channelopathy could induce a VGLUT1 synaptic disconnection on motoneurons [11,31]. Hence, the impaired vesicular-based glutamate signaling is switched to activated NMDA receptor-based signaling with a resultant NMDA persistent inward current (PIC) inducement on motoneurons [31]. These NMDA PICs could be in addition to the NaPIC ones and could be the direct result of the impairment of the monosynaptic type Ia static phase firing encoding [11,31]. It is again noteworthy that Piezo2 channels are inactivated when sustained muscle stretch occurs, and from then on, Na_v_1.1 activity transmits proprioceptive signaling to maintain regular and reliable firing [52]. However, the proposed Piezo2 channelopathy-induced switch to type II static firing and the resultant delayed or loss of Na_v_1.1 activity on type Ia fibers could be the explanation for the induced NaPICs on spinal motoneurons. These NMDA and NaPICs on spinal motoneurons could explain the exaggerated contractions and reduced range of motion due to proprioceptive terminal microinjury, as was suggested earlier [4,11,31].

The current author postulates that the aforementioned progressive loss of Na_v_1.1 activity from intrafusal proprioceptive terminals due to irreversible loss of Piezo2 input could initiate the impairment of Na_v_1.6 Na^+^ channels in the SOD1^G93A^ mouse model for ALS, as shown by Seki et al. [54]. Indeed, their computational modeling based on their observed sensory pattern disruption projected an asynchronous self-sustained motor neuron discharge [54]. Seki et al. also found their prediction to be suggestive of a novel reflex circuit-specific proprioceptive sensory abnormality in ALS [54], which could be in line with the delayed latency of MLR finding downstream in the pathomechanism in this lethal disease [5,11].

In summary, the author of this manuscript proposes based on the work of Seki et al. that the SOD1^G93A^ mutation-induced oxidative damage leading to abnormal protein synthesis could lead to Na_v_1.6 Na^+^ channelopathy in the Mes V neurons of a mouse model for ALS only after earlier primary microdamage, namely the lost function of Piezo2 and associated Na_v_ channelopathies that lead to the proposed progressive proprioceptive terminal detachment in ALS. The order of proprioceptive terminal channelopathies are suggested to represent principality and primacy in terms of the demanded mitochondrial supply for their functionality. Accordingly, it was suggested that Piezo2 is the principal one and the non-contact terminal microinjury of it could lead to the progressive non-contact dying-back injury mechanism of ALS and eventually to incompatibility with life sustainment when environmental risk factors or genetic predisposition, such as SOD1 mutation, are associated with it [4,5].

### 3.2. Autonomic Disbalance and the Painless Condition of ALS

It has been put forward that the imbalanced subthreshold cationic currents and concomitant NMDA receptor activation due to impairment of the glutamate vesicular release are to blame for the transient autonomic disbalance in ASR-induced Piezo2 channelopathy [8,31].

There is a point in ALS disease progression when functioning α-motor neurons cannot be modulated by the others as a result of the progressive death of these motoneurons [4]. This implies a more frequent ASR inducement because force production depletion is unacceptable by cognitive demand. Therefore, a chronic or more persistent “over-reaching” is needed to accomplish the desired task. Accordingly, animal research showed that ALS-inducing factors indeed sensitize sensory neurons to stress [55], which could be the result of the aforementioned “mis-wiring”. In addition, it is proposed that persistent stress granules could contribute to pathological ALS inclusions as a result of their imbalanced assembly and clearance [56]. Moreover, autonomic hyperactivity is noticed in the early phase of ALS with later hypoactivity when the disease progresses [57]. The hypoactivity of the autonomic nervous system could be indicative of the neurodegeneration downstream in reference to the over-consumed “over-reaching” response [4].

It is important to note that the absence of the theorized Piezo2 channelopathy that is associated with impairment of the glutamate vesicular release induced VGLUT1 synaptic disconnection-derived NMDA receptor activation pathway, could have relevance in the painless feature of ALS. The current author proposes that lost function of Piezo2 on type Ia fibers, due to detachment of proprioceptive terminals, also means that not only Piezo2 currents are lost (experienced during Piezo2 activation), but also subthreshold imbalanced cationic/Ca^2+^ currents as well (suggested under Piezo2 channelopathy). This means that NMDA receptor activation could be absent at the segmental spinal level. It is important to note that activated NMDA receptors are suggested as the gate controllers of DOMS [4], which is in accordance with the gate control theory of pain [58]. Moreover, spinal wide dynamic range (WDR) neurons in the dorsal horn have been long associated with this pain theory [59,60]. Indeed, NMDA receptors in association with L-type calcium currents and calcium-activated nonspecific cationic currents could activate WDR neurons [61]. This finding could be indicative that these L-type calcium currents and nonspecific cationic currents could be induced by the central terminal of the same pseudounipolar type Ia proprioceptive sensory neurons with Piezo2 channelopathy on the peripheral terminal (See Table 2). Accordingly, loss-of-function mutations in Piezo2 cause loss of pain and sensitization [62]. In addition, research is emerging that Piezo participates in L-type calcium current modulation [63]. Ca_v_1.2 and Ca_v_1.3 L-type calcium channels have been shown to influence WDR activation in the spinal dorsal horn and Ca_v_1.3 is the one responsible for initiating wind-up with the involvement of NMDA receptor activation [64]. Not to mention that the role of WDR neurons on the spinal dorsal horn is emphasized in the pain sensitization mechanism [65].

Furthermore, acid-sensing ion channel 3 (ASIC3) is also known to activate WDR neurons in the spinal dorsal horn [66], and ASIC3 activation in intrafusal type II fibers is proposed in relation to type Ia proprioceptive terminal Piezo2 channelopathy in DOMS [7]. ASIC channels have dual functions with the capability of chemo-sensing and mechano-sensing [67]. The author of this manuscript suggests that ASIC3 could be the unknown ion channel responsible for the detection of deep tissue sensation in type II fibers [68]. Correspondingly, the current author proposes that Piezo2 channelopathy induced activated NMDA receptors, subthreshold imbalanced Ca^2+^ currents, and possibly activated ASIC currents could initiate intrafusal mechanical hyperalgesia in DOMS with WDR neuron activation in the dorsal spinal horn.

However, nociceptive C-fiber contribution is also essential in DOMS, as shown by Kubo et al. [69], and is suggested by the non-contact acute compression axonopathy theory of DOMS [12,31]. Of note, that C-fiber contribution is suggested to evolve only in the secondary damage phase of DOMS [8,31]. Ota et al. showed that transient receptor potential 1 (TRPV1) ion channel contribute to DOMS as well, and even suspected that this signaling is conveyed by nociceptive type IV neurons, but certainly in the extrafusal compartment [70]. It is important to note that TRPV1 increases the responses of WDR neurons in tissue injury by affecting mechanotransmission and spontaneous pain [71]. Moreover, the crosstalk and cross-activation of all four types of hyperexcited type Ia, type II, and type IV fibers is theorized to be essential for the evolvement of DOMS [12,31].

On the contrary, in ALS, the detachment of proprioceptive terminals means lost Piezo2 function and lost ASIC3 function at the primary afferents. This also means lost NMDA receptor activation, lost subthreshold imbalanced Ca^2+^ currents, and lost ASIC currents; therefore, WDR neurons cannot be activated and the cross-activation with C-fibers cannot evolve. As a consequence, pain and mechanical hyperalgesia cannot develop either in association with the loci of the proposed intrafusal non-contact microinjuries in ALS.

## 4. Aging

One of the most challenging, but unanswered theoretical question of science is the root cause of the “primary damage” of aging [72]. Correspondingly, a quad-phasic non-contact injury model was proposed as a result of Piezo2 channelopathy, emphasizing that Piezo2 microinjury is one principal gateway to pathophysiology [9,26,73]. It is widely observed that a vast majority of neurodegenerative diseases are associated with aging, as is the case in ALS. Mitochondria take a central stage in this aging process. Accordingly, the intimate crosstalk between mitochondria, lysosomes, and the endoplasmic reticulum is essential for life maintenance, but could be also independent activators of apoptosis in response to cell stress through H_2_O_2_, cathepsins, and more importantly, Ca^2+^ [74]. It is important to note again that one suggested consequence of Piezo2 channelopathy is the leakage of subthreshold imbalanced Ca^2+^ currents when Piezo2 should be intact and inactivated in a hyperexcited state [8,11]. The current author postulates that these subthreshold imbalanced Ca^2+^ currents could disturb the intimate crosstalk between mitochondria, lysosomes, and the endoplasmic reticulum of proprioceptors, hence accelerating the aging process when it takes a chronic path. Even more importantly, the complete loss of Piezo2 currents could also irreversibly disrupt this intimate crosstalk between these organelles of proprioceptors, leading to lost homeostasis maintenance, terminally impaired transcription processes, and apoptosis, as can be the case in ALS [5].

### 4.1. Aging of Mitochondria

The steady supply of energy from the mitochondria is essential for cell and tissue function [75]. How sophisticated this energy producing symbiotic relationship of bacteria-derived mitochondria with host cells is represented in that the greater part of mitochondrial proteins are encrypted and synthesized in the host cell [76] and transported in order to be imported by the mitochondrial translocation machinery [74]. Any discord in this intimately coordinated action could lead to inadequate energy supply, loss of homeostasis, and could reduce human longevity [74]. Molecular oxygen is used for energy production, but it could be a source for reactive oxygen species as well (ROS) [74]. Therefore, the aforementioned discord could take a pathogenic turn toward excessive ROS production. This imbalance towards pro-oxidants facilitates oxidative damage [77], in a process where lipids, proteins, and nucleic acids of mitochondria and host cells will be the most exposed [74]. Chronic excessive ROS-induced oxidative damage could lead to apoptosis and is associated with aging, neurodegenerative diseases, not to mention cancer [78,79]. Accordingly, redox imbalance, dysregulation of Ca^2+^, and/or lost mitochondrial membrane potential are suggested to increase mitochondrial outer membrane permeability and activation of the mitochondrial intrinsic apoptotic pathways [74]. As a result, the aging mitochondria features morphological abnormalities [80,81], lower mitochondrial respiratory chain and oxidative phosphorylation activities [82,83], and impaired energy production [74,84,85].

In summary, aging-associated mitochondrial dysfunction is due to intrinsic mitochondrial deficiency and to the reduction of their numbers [74,85,86,87].

### 4.2. Inadequate Mitochondria Supply to Proprioceptive Terminals

It is important to highlight the difference in neural input of eccentric contractions as opposed to concentric contractions [88,89,90]. Eccentric contractions have higher force generation and lower motor unit discharge [88,90,91], hence a better energetic profile [92] than concentric contractions [89]. However, these advantages of eccentric contractions come at the price of increased proprioceptive loading of the sensory afferent terminals of the muscle spindle [4,7], not to mention increased mitochondrial loading in these terminals in order to exert the higher force generation. This also means that adequate mitochondrial trafficking and supply is critical not only in the case of motoneuron terminals, but to intrafusal proprioceptive terminals as well under unaccustomed or strenuous eccentric contractions, otherwise it is proposed to lead to TAD-like lesions or, more precisely, to Piezo2 channelopathy [8,12].

It could be learnt from chemotherapy that eccentric contraction-induced proprioceptive impairment is most likely due to inhibition of tubulin polymerization resulting from the disruption of microtubule function along the axon length [93]. Microtubules are essential for maintaining cell structure by forming the cytoskeleton, but they also have a role in mitochondrial transport. In an intact microtubule system, the efficient distribution of mitochondrial transport along neurons is important, especially to synaptic terminals [94,95,96,97]. The current author suggests that damaging eccentric contractions disrupt the proper assembly of microtubules and, even more importantly, impair proper distribution within the mitochondrial transport system. Eventually, the disruption of the respiratory chain along the axon could evolve [96], providing the base for TAD-like lesions. Bennett et al. proposed in their TAD theory that this degenerative lesion could occur in the hyperexcited highest energy demanding neuronal compartment [13], such as intrafusal proprioceptive terminals under unaccustomed or strenuous eccentric contractions [12]. As a result, fatigued and microinjured proprioceptive sensory fibers could not execute the recoil characteristics of eccentric contractions efficiently, therefore, the excess unrecoiled energy could damage the microtubule system and the respiratory chain of proprioceptive axons, leading to an improper functional mitochondrial supply at the proprioceptive terminals under an ASR, especially when the aging process comes along [4,31]. Moreover, it is known that mitochondrial proteins take part in the mitochondrial stress response [98]. Indeed, an intact cytoskeleton is essential for proper Piezo2 function and the disruption of the actin or microtubule polymerization diminishes Piezo2 currents [99,100,101,102,103]. Even more importantly, it is theorized that an inadequate mitochondrial supply and dysfunctional mitochondria could lead to Piezo2 channelopathy at the proprioceptive terminals [5,8,11,31].

In summary, dysfunctional mitochondrial energy supply could be due to extrinsic causes of inadequate mitochondrial trafficking to the proprioceptive terminals of the muscle spindle under unaccustomed or strenuous eccentric contractions, especially during aging or neurodegenerative diseases, as is suggested in ALS. It is noteworthy that mitochondrial supply has enormous buffering potential over dysfunctionality. Indicative of this mitochondrial buffering capacity, neuropathy, ataxia, and retinitis pigmentosa, also known as NARP syndrome, only evolves if 70–90% of mitochondria carry the syndrome-specific mutation. If the aforementioned mutation is represented in more than 90% of mitochondria, then an even more severe neurodegenerative disease develops, called Leigh syndrome [104]. Hence, it is not only the supply of mitochondria that matters, but also the percentage of functional ones, especially during the aging process when the percentage of dysfunctional mitochondria increases. This aforementioned availability of a functional mitochondrial supply could explain the dose-limiting fashion of this microinjury, as is suggested in Piezo2 channelopathy [8] or in its irreversible microinjury [5].

### 4.3. Redox Imbalance and Oxidative Stress Leading to Piezo2 Channelopathy

Eccentric exercise-induced ROS production was demonstrated with a temporal disassociation [105], as is proposed in the acute form of the proprioceptive terminal microinjury [12]. It is noteworthy that earlier inflammatory agents in the muscle were to blame for the source of ROS production [106]. Moreover, free radicals also act as signaling molecules to control muscle cell growth, remodeling, regeneration, and proliferation [107]. On the contrary, free radicals are prone to harm neurons due to their elevated energy demand [108,109], as the radical of nitric oxide could damage the proteins and lipids of neurons as well [110], leading to a dysfunctional neural energy supply [111] and apoptosis [112,113]. Indeed, the excessive gathering of these damaging radicals could trigger the distal degeneration of sensory neurons [13]. The non-contact acute compression axonopathy theory of DOMS also implicated such an energy supply failure at intrafusal proprioceptive terminals [37], as is proposed in TAD-like lesions [13]. A review paper presented recently explains the oxidative damage and redox imbalance in more detail in association with type Ia proprioceptive terminal microdamage [31]. Indeed, Borghi et al. showed in mice that an unaccustomed bout of intense acute exercise elevated the level of superoxide anions, lipid peroxidation, and oxidative stress in the spinal cord at the peak of DOMS [114]. Furthermore, recent research verified that intense acute swimming causes oxidative stress in the spinal cord and the resultant redox imbalance had a role in nuclear factor-kappa B (NF-κB) activation [115]. Not surprisingly, NF-κB inhibitors could diminish superoxide anion production, lipid peroxidation, and oxidative stress [114]. It is noteworthy that astrocytes and microglia could also increase ROS production under pathological conditions [4,114,116,117]. It was proposed earlier that the hyperexcitation of intrafusal primary proprioceptive terminals with leakage at Piezo2 is such a pathological condition, in a similar fashion as TAD-like lesions [8,31].

In line with the aforementioned, platinum analogue chemotherapeutic drug-induced ROS production contributes to peripheral neuropathy [118], with NF-κB pathway activation [119]. The ROS derived NF-κB activation may also have a role in muscle cells as well, with associated NGF upregulation on them [120]. This upregulation of NGF is facilitated by B_2_ bradykinin-induced NF-κB activation in muscle cells [120]. Of note, bradykinin has a known function of upregulating Piezo2 currents [15,120,121], hence could contribute indirectly to the proposed Piezo2 microinjury [31].

In brief, the pathological hyperexcitation of the proprioceptive afferent terminals induces ROS production in association with neuroinflammation and neural activation in the spinal cord due to this proposed transient Piezo2 channelopathy [31], in addition to ROS generation in muscle cells. Accordingly, evidence is on the rise as to how redox dysregulation could contribute to the ALS pathomechanism [122], where the microinjury of the Piezo2 is suggested to be irreversible.

## 5. ALS Genes and the Functional Loss of Piezo2

It is important to place ALS genes and their genetic mutations on the aforementioned pathomechanistic roadmaps of the proposed Piezo2 channelopathy, both downstream and upstream. Correspondingly, ALS genes are theorized to be involved in the functional maintenance of the proprioceptive terminal Piezo2 ion channel downstream and regeneration of it upstream.

The genetic background of ALS is heterogenous. The most used classification of genes associated with ALS is as follows: major, minor, and overlapping genes. Major ALS genes have been well-investigated in contact with the disease, and their variants are known to contribute to the development of ALS. Minor or candidate ALS genes have been implied in the disease processes, but their exact role is far from entirely known in the development of ALS. Other neurodegenerative or neuromuscular diseases may show a phenotypic overlap with ALS, thus genes associated with those diseases might be important to consider when investigating the genetic background of ALS to provide a differential diagnosis.

ALS theories are summarized in the review of Robberecht and Philips as follows: excitotoxicity, astroglia and/or microglia dysfunction, oxidative stress, mitochondria dysfunction, endoplasmic reticulum stress, defects in RNA processing, growth factor abnormalities, defects in axonal transport, metabolic alterations, and accumulation of protein aggregates [123]. ALS genes could be sorted into seven groups based on the aforementioned non-contact dying-back injury mechanism theory of ALS that are overlapping the aforementioned sporadic ALS theories and describe their potential pathomechanistic role in the aforementioned autologous Piezo2 microinjury mechanism.

### 5.1. Genes Related to Intact Microtubules, Actin Stability, and Intracellular Organelle Trafficking

The preserved actin and microtubule system are essential for an intact cytoskeleton, not to mention along the axon length in order to maintain efficient distribution of mitochondrial transport, especially at synaptic terminals [94,95,96,97]. Intrafusal Piezo2-containing proprioceptive terminals are suggested to be compartments that are in heavy need of adequate mitochondrial supply under hyperexcitation due to unaccustomed or strenuous eccentric contractions, not to mention under allostatic stress. Inadequate mitochondrial transport along proprioceptive axons and supply of the terminals could result in more susceptibility to proprioceptive Piezo2 terminal microdamage. Indeed, an intact cytoskeleton is needed for Piezo2 mechanotrasduction and the disruption of microtubules decreases Piezo2 currents [103]. In summary, unaccustomed or strenuous eccentric contractions under allostatic stress during the aging process could make the proprioceptive terminal Piezo2 channelopathies irreversible, if ALS mutations are present downstream of the aforementioned non-contact injury mechanism in ALS.

### 5.2. Genes Related to Mitochondrial Functional Maintenance

It is hypothesized that mitochondria could become dysfunctional under unaccustomed or strenuous loading of eccentric contraction-induced ASR time window, leading to Piezo2 channelopathy [8]. However, ALS gene mutations could make these channelopathies irreversible, especially when the aging process comes along.

### 5.3. Genes Related to Stress Resilience and Oxidative Damage Protection

Unaccustomed or strenuous eccentric contraction under allostatic stress could microdamage proprioceptive terminal Piezo2 channels, and oxidative damage is part of this picture [12,31]. Hence, the proteins that are involved in stress buffering and oxidative damage protection in the aforementioned pathomechanism are essential in Piezo2 functional maintenance, especially during the aging process when the microinjury propensity of these Piezo2 channels are increased. Mutation of the superoxide dismutase 1 (SOD1) gene is a good example.

SOD1 is a protein coding gene that binds copper and zinc ions and is responsible for offsetting free superoxide radicals. It also has an antibacterial and antifungal activity according to the GeneCards database (www.genecards.org, accessed on 13 January 2023). A mutation of this gene could contribute to excessive oxidative damage leading to irreversible Piezo2 microinjury and lost functionality. It is noteworthy that in SOD1^G93A^ transgenic mice, the type Ia and II intrafusal proprioceptive terminals go through significant changes, even in the asymptomatic stage of ALS [1]. Interestingly, these alterations come along with simultaneous changes in α-motor neurons [1]. However, it is not surprising since the suggested initiating non-contact injury, namely irreversible Piezo2 channelopathy on primary proprioceptive terminals, develops over the monosynaptic stretch reflex arc between proprioceptive type Ia sensory fibers and α-motor neurons, and α-motor neuron terminals are also heavily energized by SOD1-containing mitochondria.

It is noteworthy that the loss of proprioceptive terminal Piezo2 function also means the silent loss of proprioception as the first injury phase of the quad-phasic non-contact injury model [9,26,73]. Sábado et al. reported that large DRG proprioceptors accumulate misfolded SOD1 in association with activated microglia and the degenerative process [124]. They translated their finding as transsynaptic propagation of SOD1 misfolding from ventral motoneurons to DRG neurons in a prion-like mechanism through a monosynaptic connection [124]. However, the current author proposes exactly the opposite direction of the propagation based on the aforementioned non-contact microinjury mechanism. Accordingly, even if the proprioceptive ending alterations come along with simultaneous changes on α-motor neurons, the initiating microdamage could be on Piezo2-containing intrafusal proprioceptive terminals. Moreover, prion-like propagation is suggested to be propelled by eccentric contractions towards the CNS in the form of damaging currents [4].

### 5.4. Genes Affecting Vesicle-Mediated Assembly, Transport, and Release

The primary microinjury of these Piezo2 proprioceptive fiber terminals is suggested to be associated with impaired glutamate vesicular release [5,8,31]. ALS genes that are affecting vesicle-mediated assembly, transport, and release could lead to susceptibility to Piezo2 microinjury and irreversible terminal detachment, especially during the aging process when these ion channels and their compartmental environment are more unstable structurally.

### 5.5. Genes Affecting Transcription, Development, Growth, and Apoptosis

The primary microinjury of the Piezo2-containing type Ia proprioceptive fibers could activate transcription pathways that are the basis for the aforementioned growth, healing, regeneration, and remodeling processes. The complete loss of Piezo2 function could lead to the loss of these activated and dysregulated transcription pathways eventually, which is incompatible with life sustainment and leads to apoptosis and death, as is proposed in the case of ALS [5]. TARDBP mutation is a good example, since TAR DNA binding protein-43 (TDP-43) controls non-coding and protein-coding RNA splicing, including relevant proteins of neurodegenerative diseases, and contributes to the survival of neurons as well [125,126]. Moreover, it is also involved in transcription control in order to maintain mitochondrial homeostasis [127] and contributes to cell survival during oxidative insult-induced cellular stress [128,129]. It participates in skeletal muscle formation and regeneration as well [130]. However, a mutation of this gene could contribute to dysfunctional transcription processes, even mitochondrial, and reduces survival due to oxidative stress when Piezo2 channelopathy is present. Interestingly, in contrast with SOD1^G93A^ transgenic mice where intrafusal proprioceptive terminals degenerate simultaneously with monosynaptically connected motoneuron terminals, the TDP43^A315T^ transgenic mice show a different pattern where type Ia and type II sensory endings degenerate earlier than α-motoneuron terminals [1]. According to the current author, this difference is indicative that the initiating pathomechanistic non-contact injury mechanism is on proprioceptive terminals in ALS, and this initiated irreversible intrafusal proprioceptive terminal degenerative detachment is the reason why ALS is considered a painless condition, as mentioned earlier.

### 5.6. Protein Metabolism, Degradation, and D-Amino Acid Removal

Resistance exercise with eccentric contractions enhances the unfolded protein response (UPR) [131]. It is postulated that in DOMS, which is proposed to involve a transient Piezo2 channelopathy as the primary damage [8,31], the activation of the UPR pathway and protein degradation are within homeostasis and is initially suggested to be a protective mechanism against unwanted physical currents [4]. Accordingly, full functional regeneration will evolve in intrafusal microdamaged proprioceptive terminals [4]. On the contrary, the UPR and protein degradation are in a state of imbalance in ALS [4], probably due to the mutations of the involved genes when Piezo2 channelopathy and their functional loss leads to neuromuscular detachment in the proprioceptive terminals of the muscle spindle.

### 5.7. Genes Affecting Neuroinflammation

In the acute type of the proposed Piezo2 channelopathy-derived neuroinflammation, as is suggested in DOMS [12,31], the critical role of Hsp70/TLR4/Interleukin-6, TLR4/Myd88, and TNF-α pathways are demonstrated [114,132,133]. Furthermore, the TLR4/Myd88 signaling pathway also activates the NF-κB pathway [114,132]. Accordingly, when the suggested Piezo2 microinjury evolves to be irreversible with associated detachment of intrafusal proprioceptive neuromuscular junctions in ALS, then the activated NF-κB pathway still sustains neuroinflammation. In addition, they contribute to the non-resolving progressive impairment of the proprioceptive circuitry in ALS. Hence, the mutation of ALS genes that are affecting neuroinflammation has relevance in the ALS pathomechanism.

## 6. Conclusions

The current manuscript postulates that Piezo2 channelopathy is a principal transcription activator in proprioceptive terminals. It has relevance in the ALS pathomechanism with activated and dysregulated transcription, but impediment of completing the transcription process. This constant transcription activation could provide access to underlying pathogenic gene variants and letting the cell-type-specific noncoding DNA mutations become more apparent in ALS. The author proposes that ALS genes are associated with the Piezo2 channelopathy mechanism both downstream and upstream, and their mutations could explain the non-contact dying-back injury mechanism theory of ALS. Moreover, microinjury of the Piezo2 ion channel could be the aforementioned “primary damage”. This is in line with a recent concept that Piezo2 channelopathy is one principle gateway to pathophysiology, and it is also depicted in their quad-phasic non-contact injury model where Piezo2 channelopathy is the primary damage phase [26]. In addition, the progressive loss of Piezo2 functionality is proposed to not be compatible with life sustainment [5].

## Figures and Tables

**Table 1:** The quad-phasic non-contact injury model adapted to ALS [26].

Piezo2 Microdamage Induced Quad-Phasic Non-Contact Injury Model Adapted to ALS [26]				
**Environmental Factors**		**Primary Injury Phase**		**Genetic Predisposition**
	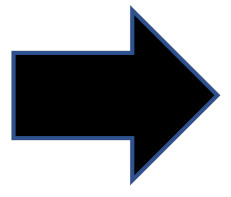	Repetitive eccentric muscle contractions	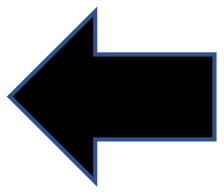	
		Damaging unaccustomed or strenuous exercise-based fatigue-induced acute stress response due to cognitive demand		
	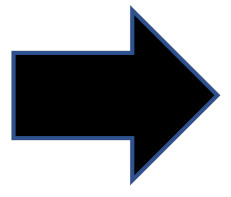	Stress-derived energy depletion of the proprioceptive terminal mitochondria and/or inadequate mitochondrial trafficking to the proprioceptive terminals	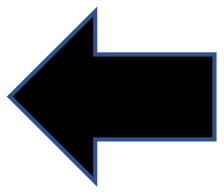	
		Painless irreversible mechano-energetic microdamage of intrafusal proprioceptive Piezo2 and extrafusal motoneuron death		
	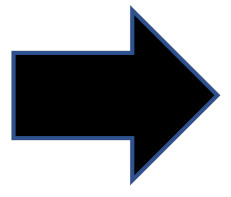	**Secondary Injury Phase**	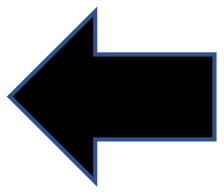	
		Harsher tissue damage due to the loss of intrafusal Piezo2 functionality without extrafusal nociceptive fiber contribution		
	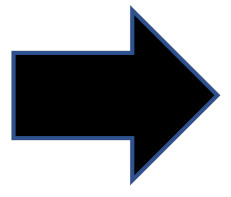	**Tertiary Injury Phase**	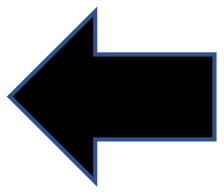	
	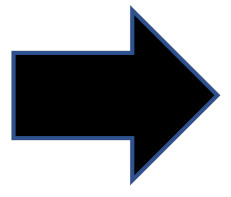	Chronic neuroinflammation or ganglionopathy Progressive loss of intrafusal Piezo2 Progressive loss of extrafusal motoneurons Lost cross-talk between Piezo1 and Piezo2, leading to muscle atrophy	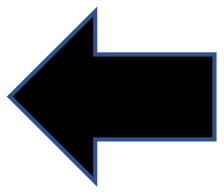	
		**Quadric Injury Phase**		
	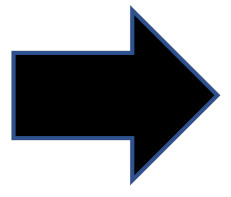	Aging or non-resolving neuroinflammation-induced irreversible Piezo2 microinjury or the augmentation of the former Piezo2 channelopathy to the level of irreversibly lost Piezo2 functionality, leading eventually to death	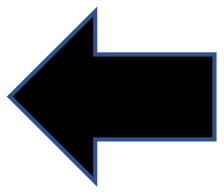	

**Table 2:** The primary damage mechanism in ALS and DOMS *, partially adapted from exercise-induced microdamage [7,31].

	Amyotrophic Lateral Sclerosis (ALS)		Delayed Onset Muscle Soreness (DOMS)	
**Primary intrafusal injury phase**	YES	Irreversible intrafusal proprioceptive terminal microdamage	YES	Transient intrafusal proprioceptive terminal microdamage
	NO	Lost NMDA receptor activation on spinal dorsal horn due to irreversibly lost glutamate vesicular release on type Ia proprioceptive neurons	YES	NMDA receptor activation on spinal dorsal horn due to impairment of the glutamate vesicular release on type Ia proprioceptive neurons
	NO	Lost L-type calcium currents and nonspecific cationic currents in spinal dorsal horn due to lost Piezo2 functional on peripheral proprioceptive terminals	YES	L-type calcium currents and nonspecific cationic currents in spinal dorsal horn due to peripheral proprioceptive terminal Piezo2 channelopathy induced subthreshold imbalanced calcium currents
	NO	Wide dynamic range (WDR) neuron activation	YES	WDR neuron activation
**Soreness condition**	Considered as a painless disease		DOMS lasting up to 7 days	

* Both ALS and DOMS are suggested to contain intrafusal microdamage on proprioceptive terminals. However, in the case of ALS, it is proposed to be irreversible. On the contrary, the microdamage on proprioceptive terminals is transient in the case of DOMS. Hence, the progressive loss of intrafusal proprioceptive Piezo2 function will lead to neurodegeneration and eventually to death in ALS.

## Data Availability

Not applicable.
